# Interaction of the tracheal tubules of *Scutigera coleoptrata* (Chilopoda, Notostigmophora) with glandular structures of the pericardial septum

**DOI:** 10.3897/zookeys.510.8644

**Published:** 2015-06-30

**Authors:** Gero Hilken, Gregory D. Edgecombe, Carsten H.G. Müller, Andy Sombke, Christian S. Wirkner, Jörg Rosenberg

**Affiliations:** 1 Central Animal Laboratory, University Clinic of Essen, Hufelandstraße 55, D-45122 Essen, Germany; 2 The Natural History Museum, Cromwell Road, London SW7 5BD, UK; 3 Zoological Institute and Museum, University of Greifswald, Soldmannstrasse 23, D-17489 Greifswald, Germany; 4 Department of Neuroscience, University of Arizona, 1040 E 4th Street, PO Box 210077, Tucson, AZ 85721, USA; 5 Allgemeine & Spezielle Zoologie, Institut für Biowissenschaften, Universität Rostock, Universitätsplatz 2, D-18055 Rostock, Germany; 6 Am Kützelbach 3, D-59494 Soest, Germany

**Keywords:** Centipedes, electron microscopy, tracheal system, aggregated recto-canal epidermal glands, respiration

## Abstract

Notostigmophora (Scutigeromorpha) exhibit a special tracheal system compared to other Chilopoda. The unpaired spiracles are localized medially on the long tergites and open into a wide atrium from which hundreds of tracheal tubules originate and extend into the pericardial sinus. Previous investigators reported that the tracheal tubules float freely in the hemolymph. However, here we show for the first time that the tracheal tubules are anchored to a part of the pericardial septum. Another novel finding is this part of the pericardial septum is structured as an aggregated gland on the basis of its specialized epithelium being formed by hundreds of oligocellular glands. It remains unclear whether the pericardial septum has a differently structure in areas that lack a connection with tracheal tubules. The tracheal tubules come into direct contact with the canal cells of the glands that presumably secrete mucous substances covering the entire luminal cuticle of the tracheal tubules. Connections between tracheae and glands have not been observed in any other arthropods.

## Introduction

The history of research on tracheae of *Scutigera coleoptrata* extends back to the 19^th^ century. Meinert (1868, 1883) denied the existence of tracheae and thought the dorsally located spiracles (Haase 1884: “stomata”) are the openings of sticky glands. Pagenstecher (1878), Tömösváry (1881, 1883a, b), Sinclair (1891, 1892), Voges (1882, 1916), Haase (1883, 1884, 1885), and Chalande (1885) were the first to recognize the dorsally situated tracheal systems as the real respiratory organs. Fine structural investigations on the respiratory system and the tracheal tubules were conducted by Prunescu and Prunescu (1996) and Hilken (1997, 1998). The entity of tracheal tubules has often been termed “lungs”, tracing back to Chalande (1885: “appareil pulmonaire“). Dubuisson (1928) studied the interaction of both tracheal and circulatory systems with regard to active ventilation. He called the space around the tracheal system – bounded by a septum – a ‘cavité péricardique’. The tracheal system of Notostigmophora consists of an unpaired, non-closable spiracle that opens into a wide atrium. From there, hundreds of short tracheal tubules originate and extend into the pericardial sinus. Up to now, it has been thought that the tracheal tubules end blindly and float freely in the hemolymph space that is surrounded by the pericardial septum (e.g. Sinclair 1892, Dubuisson 1928, Hilken 1997, 1998). However, more recent illustrations by Wirkner and Pass (2002: Fig. 3A, p. 198) and Wirkner et al. (2013: Fig. 14.9b, p. 358) indicated that, even though not further addressed in their anatomical descriptions, the tracheal tubules may indeed be in close contact with the pericardial septum.

During recent years, we focused on tracheal systems (Hilken 1997, 1998, Rosenberg 2009) and on comparative investigations of epidermal glands in Chilopoda with regards to their structure and phylogenetic significance (e.g. Hilken et al. 2005, 2011, Hilken and Rosenberg 2006, 2009, Müller et al. 2003, 2009, 2014). In the present study on *Scutigera coleoptrata*, we found an unexpected connection between the tracheal tubules and associated glands using TEM techniques. Here, we describe for the first time that in *Scutigera coleoptrata* (1) the distal tips of the tracheal tubules are anchored in a part of the pericardial septum, (2) this specialized epithelium of the pericardial septum consists exclusively of glandular units, and (3) there are profound interactions between tracheae and the glandular units.

## Material and methods

Specimens of *Scutigera coleoptrata* (Linnaeus, 1758) were reared in glass boxes filled with 1 cm soil substrate and moistened tissues and fed with *Drosophila* sp. and *Musca* sp. Animals were anesthetized with CO_2_, subsequently fixed, cut along the tergite edges, and preserved as described below.

For scanning electron microscopic (SEM) investigations of the tracheal tubules, whole specimens were fixed in ethanol (70%). Tracheae were macerated using pepsin. Optimal results were obtained with a solution of 1–2 g pepsin in 100 ml HCl (37%, Hilken 1994, 1998). The complex of tracheal tubules was dehydrated through a graded series of ethanol, critical-point dried, coated with gold, and studied with a CAMSCAN DV4.

For light microscopic (LM) and transmission electron microscopic (TEM) investigations, segments of *Scutigera coleoptrata* were fixed in phosphate buffered paraformaldehyde (4%, pH 7.2), containing 15% saturated picric acid and 0.08% glutaraldehyde. They were postfixed with 1% OsO_4_ in the same buffer and, after alcohol dehydration, embedded in Epon. Semithin sections (0.5–1 µm) were stained with 1% toluidine blue in a solution of 1% sodium tetraborate. Sections were studied using a DMSL-Leica microscope. Ultrathin sections were stained with uranyl acetate and lead citrate and studied using a ZEISS EM 902 A electron microscope.

## Results

**Figure 1.**
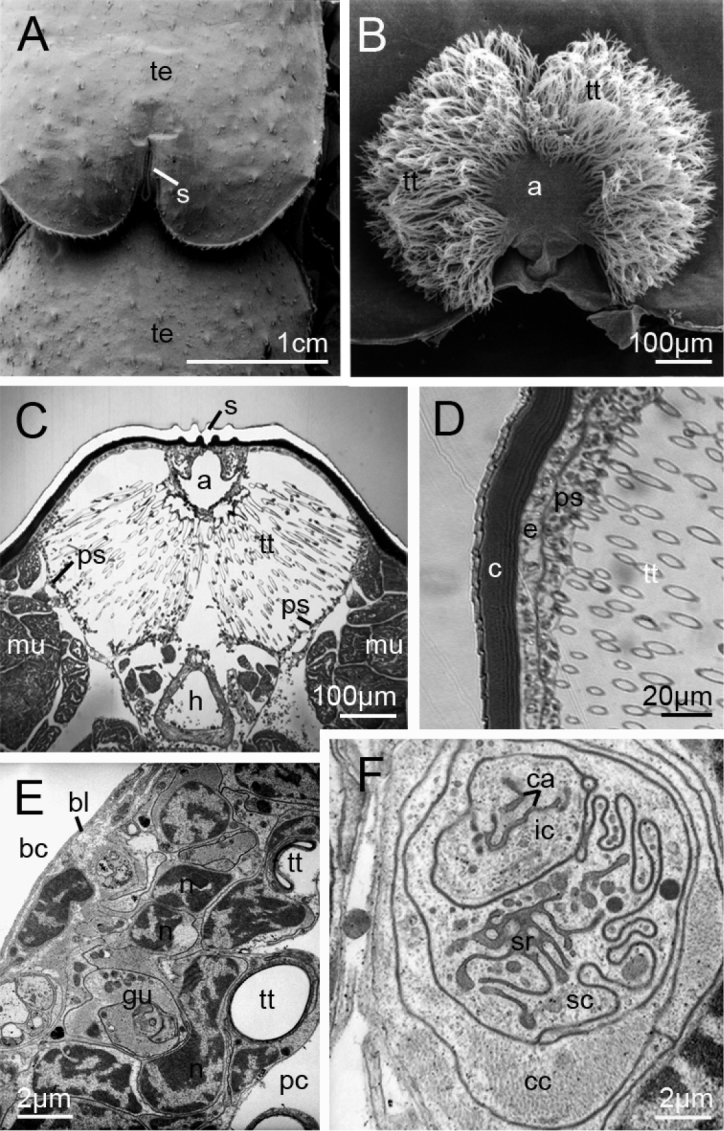
Tracheal system and pericardial septum of *Scutigera coleoptrata*. **A** Spiracle (stomata) situated dorsomedially on long tergite 4 (SEM) **B** Tracheal system with its atrium and hundreds of tracheal tubules (SEM) **C** Cross-section of tracheal system with its tracheal tubules within the pericardial sinus. The sinus is surrounded by the specialized part of the pericardial septum (LM) **D** Inset showing details of the tergal cuticle with epidermis and the pericardial septum. The tracheal tubules extend into the epithelium of the pericardial septum (LM) **E** Overview of the epithelium of the pericardial septum with endings of two tracheal tubules and nuclei of glandular units (TEM) **F** Detail of a glandular unit within the pericardial septum consisting of a secretory cell, an intermediary cell, and a canal cell. In the secretory cell, parts of the microvilli of the secretory reservoir are visible, as are parts of the canaliculi system in the intermediary cell. *a* atrium, *bc* body cavity; *bl* basal lamina, *c*, cuticle, *ca* canaliculi system, *cc* canal cell, *ic* intermediary cell, *e* epidermis, *gu* glandular unit, *h* dorsal heart, *mu* body muscle; *n* nuclei of glandular unit cells, *pc* pericardial cavity; *ps* pericardial septum, *s* spiracle, *sc* secretory cell, *sr* reservoir of the secretory cell, *te* tergite, *tt* tracheal tubules

**Figure 2.**
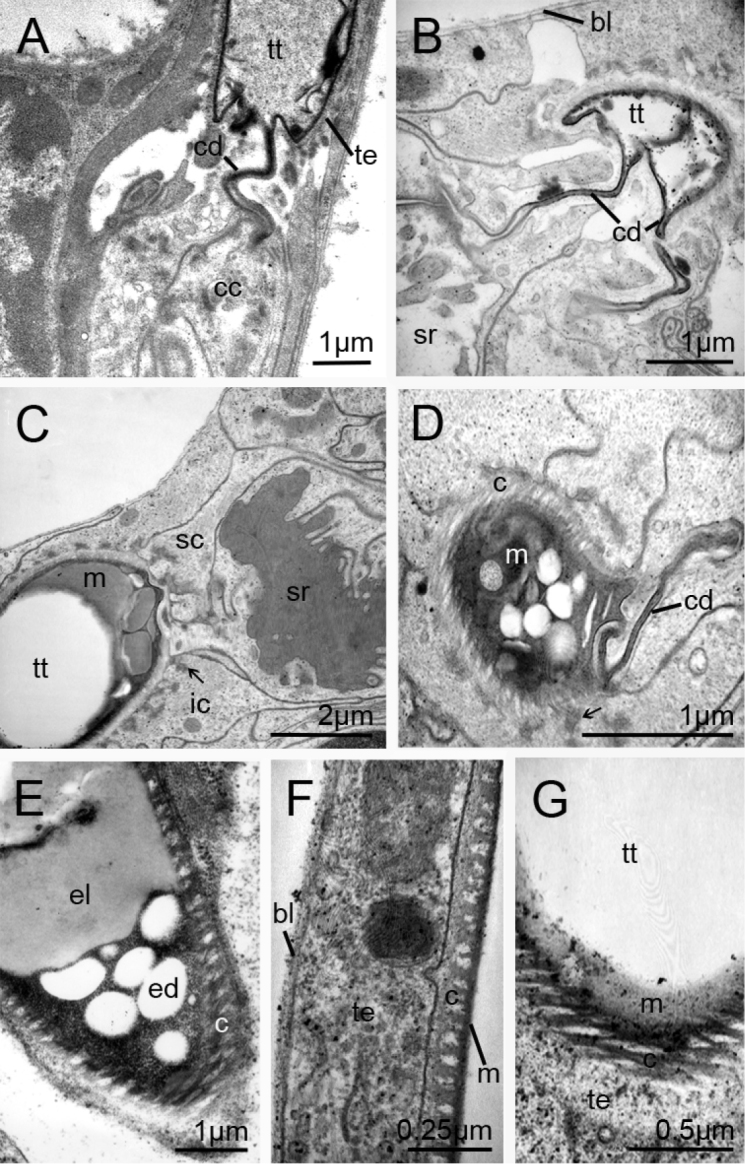
Pericardial glands and mucoid substances. **A** Longitudinal section of a connection of the tip of a tracheal tubule and a canal cell of a glandular unit **B** Cross section of a connection between a tracheal tubule and a canal cell of a glandular unit **C** Tracheal tubule in the vicinity of a glandular unit. The tracheal tubule is filled with mucoid substances **D** Cross section of the ending of a tracheal tubule surrounded by the epithelium of a glandular unit. The tracheal lumen is filled with different mucoid substances **E** Longitudinal section of a tracheal tubule near the pericardial septum filled with electron-dense and electron-lucent mucoid substances **F** Part of a longitudinal section of a tracheal tubule. The specialized cuticle is covered by mucoid substances **G** Oblique section of a tracheal tubule. The tracheal cuticle is covered by a distinct mucoid substance. *bl* basal lamina; *c* cuticle; *cc* canal cell; *cd* cuticular duct of the canal cell; *ed* electron-dense fraction of mucous; *el* electron-lucent fraction of mucus; *ic* intermediary cell; *m* mucoid substance; *sc* secretory cell, *sr* secretory reservoir; *te* tracheal epithelium; *tt* tracheal tubules; arrows, presumed interdigitations between tracheae and glandular compartment.

The unpaired openings or spiracles of the tracheal system of *Scutigera coleoptrata* are located dorsomedially on each of the seven long tergites (Fig. 1A). Each of the non-closeable spiracles (Fig. 1B) opens into a wide atrium (Fig. 1B–C). From the wall of the atrium, hundreds of short tracheal tubules originate and extend into the pericardial sinus (Fig. 1B–C). The tracheal tubules traverse the hemolymphatic space up to the epithelium of the distinct pericardial septum (Fig. 1C–E). Here, each tracheal tubule is anchored to the specialized epithelium of the pericardial septum (Figs 1E, 2A–D).

In *Scutigera coleoptrata* the length of the tracheal tubules ranges from 150–200 µm (Fig. 1B). The flat and single-layered epithelium of the tracheal tubule is thin (about 0.2–1.8 µm). A basal lamina is developed (Fig. 2F). The epithelium is covered by a specialized, extremely thin cuticle of about 0.1 µm (Fig. 2E–G). An endocuticle is covered by helically arranged chitin fibers, forming a one- or two-layered fiber network (Fig. 2E–G). Taenidia are not developed over the whole length of the tracheal tubules.

The pericardial septum arises from the atrium, partly accompanies the tergal epidermis, and delimits the hemolymph sinus against the body muscles (Fig. 1C, D). We can only provide light microscopic images from this area and the description is thus preliminary. This epithelium forms a hose-like sinus that houses all tracheal tubules. The heart lies ventral to this hemolymph sinus (Fig. 1C).

The polarity of the pericardial septum is characterized by the development of a distinct basal lamina against the body cavity (Fig. 1E). The opposite apical side of the epithelium is characterized by interactions with the tracheal tubules. Cellular junctions are not found, the contact being developed mainly as a small epithelial layer. In the areas in contact with the tips of the tracheal tubules, the specialized, apparently pseudostratified epithelium consists exclusively of hundreds of glandular units (Fig. 1E). Each glandular unit is composed of a secretory cell, an intermediary cell, and a canal cell (Fig. 1F). The tip of each tracheal tubule is anchored to the epithelium of the pericardial septum and comes into direct contact with the canal cell of each glandular unit (Fig. 2A–B). Thus, substances are secreted directly via the canal cells into the tracheal tubules. At the point of contact, presumed cellular interdigitations are formed by the surrounding cells (arrows, Fig. 2C). The lumen of the terminations of the tracheal tubules is often completely filled by mucoid substances (Fig. 2C–G). Two mucoid substances are discernible at the outermost tips of the tracheal tubules. An electron-dense fraction that contains several electron-lucent droplets is covered by an electron-lucent fraction (Figs 2C–E). A more or less dense layer of electron-lucent mucoid substances covers the cuticle along the tracheal tubules (Fig. 2F–G).

## Discussion

We are now able to resolve previous confusion on the length and connectivity of tracheal tubules of scutigeromorph centipedes. Whereas the majority of reports thus far considered tracheal tubules to end freely in the tracheal sinus compartments (e.g. Sinclair 1892, Dubuisson 1928, Prunescu and Prunescu 1996, Hilken 1997, 1998), we can now show that in *Scutigera coleoptrata* the tracheal tubules are anchored in a part of the pericardial septum. Our observations add further evidence to conclusions drawn from illustrations by Wirkner and Pass (2002) and Wirkner et al. (2013). Other findings described herein are also new, such as (1) the epithelium of the pericardial septum consists of hundreds of glandular units, and (2) the glands of the pericardial septum open into the tracheal tubules. It could be shown that the tips of the tracheal tubules are pierced by the conducting canals of numerous specialized glands. These glandular units are aggregated in a fashion similar to the class of aggregated epidermal glands (see below).

The structure and position of scutigermorph (notostigmophoran) tracheal systems are unique among Chilopoda (Hilken 1997, 1998, Hilken et al. 2011) and have been proposed as constitutive features (apomorphies) of this group (e.g., Borucki 1996, Edgecombe and Giribet 2004). In comparison to their sister group, the Pleurostigmophora, representatives of Notostigmophora possess unpaired, dorsal spiracles localized at the posterior edges of the seven long tergites. The respiratory organ is formed by hundreds of short tracheal tubules which formerly have been described as not having any contact with the organs of oxygen consumption, i.e. muscles or the nervous system (Prunescu and Prunescu 1996, Hilken 1997, 1998). The tracheal tubules are situated within dorsal compartments of the pericardial sinus. Therefore, they are surrounded by hemolymph. Interactions between tracheal tubules and hemocytes have previously been observed (Hilken et al. 2003). The hemolymph is enriched with the respiratory pigment hemocyanin (e.g., Mangum et al. 1985, Kusche et al. 2003). The tracheal tubules are strengthened by helically arranged chitin fibers, forming a one- or two-layered fiber network, whereas taenidia are not developed (Prunescu and Prunescu 1996, Hilken 1997, 1998). Because of these findings and the different position of the stigmata, it has frequently been inferred that the tracheae of notostigmophorans are not homologous to those of Pleurostigmophora and that they might be of an independent origin (Haase 1884, Dohle 1988, 1997, Minelli 1993, Hilken 1997, 1998).

Recently, there has been a particular increase in knowledge of epidermal glands of various degrees of organization in Chilopoda. It is possible to distinguish four classes of epidermal glands: 2-cell-glands (composed of a single secretory cell and a single canal cell), 3-cell-glands (composed of a proximal secretory cell, an intermediary cell, and a distal canal cell), 4-cell-glands (composed of a proximal secretory cell, an intermediary cell, a distal canal cell, and a proximal canal cell), and 5-cell glands (composed of two secretory cells, an intermediary cell, a distal canal cell, and a proximal canal cell) (see Table 2 in Müller et al. 2014). According to the terminology introduced by Hilken et al. (2005), Rosenberg (2009), Müller et al. (2009), and Rosenberg et al. (2011), three classes of epidermal glands can be distinguished depending on their structural complexity: (1) solitary epidermal glands, (2) aggregated epidermal glands (e.g. maxillary organ gland, vesicular gland; Hilken and Rosenberg 2009, Hilken et al. 2005), and (3) compound epidermal glands (maxilla-I-gland, accessory glands; Carcupino 1996, Hilken et al. 2005, Hilken and Rosenberg 2006, Rosenberg 2009, Rosenberg et al. 2011). The diversity and classification of oligocellular epidermal glands were modified, including a classification into recto-canal and flexo-canal epidermal glands, by Müller et al. (2009, 2014).

Many features indicate that the glands in question strongly resemble aggregated recto-canal glands in the epidermis. These include the following: (1) the structure of the glands associated with the tracheae; (2) the universal tricellular construction of the glandular units (consisting of a canal cell, an intermediary cell, and a secretory cell); (3) the presence of an intermediary cell surrounding an almost non-cuticularized duct; and (4) the independent release of a secretion of every glandular unit into the tracheal tubules via its own duct. Thus, an epidermal origin of the tracheae-associated glands cannot be excluded. In no other arthropods are connections between tracheae and multicellular glands observed. Future studies are needed to unravel the cellular diversity, functional complexity, distribution, and organization of the pericardial septum as a whole.

The closely adjoined glandular units release their secretion via canal cells into the connected tracheal tubules. It is likely that each secretory gland secretes mucous substances into the tracheal tubules which cover the luminal side of the cuticle of each tracheal tubule along its entire extension. Sundara Rajulu (1971) detected acid mucopolysaccharides and collagen in the tracheal tubules of *Thereuopoda longicornis* (Scutigeridae, Thereuoneminae) by histological staining. It is possible that these mucopolysaccharides correspond to the mucoid substances observed here on the cuticle of the tracheal tubules in *Scutigera coleoptrata*. It can be assumed that the mucoid layer might support the uptake and/or diffusion of oxygen over the tracheal cuticle and the epithelium into the hemolymph. Here, the respiratory protein hemocyanin serves as a transport molecule of oxygen into the body fluid. Hemocyanins are present in the hemolymph of various myriapod species and have been identified in *Thereuopoda longicornis* (Sundara Rajulu 1969) and *Scutigera coleoptrata* (Mangum et al. 1985), as well as in various Spirostreptidae (Diplopoda) (Jaenicke et al. 1999, Kusche and Burmester 2001, Hagner-Holler 2004). The hemocyanin of *Scutigera coleoptrata* displays a low oxygen affinity and allosteric behavior with very high cooperativity. It is thus an efficient oxygen carrier with considerable functional plasticity (Mangum 1985, Mangum and Goddet 1986, Burmester 2001, 2002, Kusche et al. 2003). Facilitated uptake and/or diffusion of oxygen into the body and subsequent uptake and transport within the hemolymph provide for a passive exhaust ventilation of the tracheal tubules. Thus, we assume that the mucoid substances secreted by the aggregated epidermal glands of the pericardial septum are important in notostigmophoran respiratory physiology.
